# Kinetics of the Gas-Phase Reactions of *syn*- and *anti*-CH_3_CHOO Criegee Intermediate Conformers with SO_2_ as a Function of Temperature and Pressure

**DOI:** 10.1021/acs.jpca.4c00199

**Published:** 2024-03-29

**Authors:** Rachel E. Lade, Lavinia Onel, Mark A. Blitz, Paul W. Seakins, Daniel Stone

**Affiliations:** † School of Chemistry, University of Leeds, Woodhouse Lane, Leeds LS2 9JT, U.K.; ‡ National Centre for Atmospheric Science, University of Leeds, Woodhouse Lane, Leeds LS2 9JT, U.K.

## Abstract

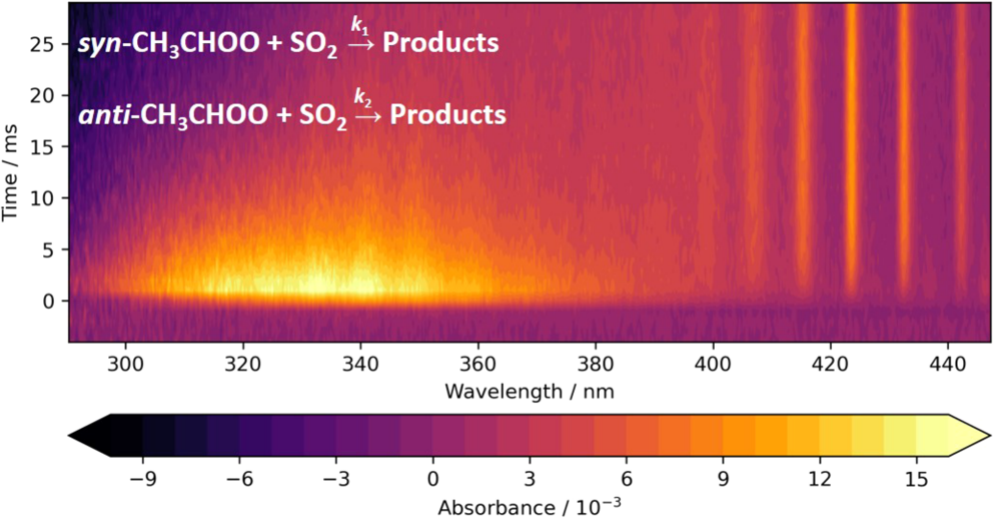

Kinetics of reactions between SO_2_ and CH_3_CHOO Criegee intermediate conformers have been measured at temperatures between 242 and 353 K and pressures between 10 and 600 Torr using laser flash photolysis of CH_3_CHI_2_/O_2_/N_2_/SO_2_ gas mixtures coupled with time-resolved broadband UV absorption spectroscopy. The kinetics of *syn*-CH_3_CHOO + SO_2_ are pressure-dependent and exhibit a negative temperature dependence, with the observed pressure dependence reconciling apparent discrepancies between previous measurements performed at ∼298 K. Results indicate a rate coefficient of (4.80 ± 0.46) × 10^–11^ cm^3^ s^–1^ for the reaction of *syn*-CH_3_CHOO with SO_2_ at 298 K and 760 Torr. In contrast to the behavior of the *syn*-conformer, the kinetics of *anti*-CH_3_CHOO + SO_2_ display no significant dependence on temperature or pressure over the ranges investigated, with a mean rate coefficient of (1.18 ± 0.21) × 10^–10^ cm^3^ s^–1^ over all conditions studied in this work. Results indicate that the reaction of *syn*-CH_3_CHOO with SO_2_ competes with unimolecular decomposition and reaction with water vapor in areas with high SO_2_ concentration and low humidity, particularly at lower temperatures.

## Introduction

The chemistry of Criegee intermediates (R_2_COO) exerts potential impacts on air quality and climate through their involvement in atmospheric oxidation processes, and there has been considerable interest in the potential production of sulfate aerosols resulting from the reactions of Criegee intermediates with sulfur dioxide (SO_2_) in the gas phase. Production of Criegee intermediates in the atmosphere occurs following the oxidation of unsaturated volatile organic compounds (VOCs) by ozone (O_3_), with the Criegee intermediate initially produced with high internal energy.^1^ Collisional stabilization of the nascent Criegee intermediate occurs in competition with unimolecular decomposition, leading to the production of stabilized Criegee intermediates (SCIs), which can participate in a range of processes, including reactions with SO_2_.^1,2^

For the reaction of the simplest SCI, CH_2_OO, with SO_2_, there is now general consensus regarding the kinetics at room temperature, with a current IUPAC recommendation of (3.7_–0.40_^+0.45^) × 10^–11^ cm^3^ s^–1^ at 298 K.^3^ The kinetics of CH_2_OO + SO_2_ have been demonstrated to be independent of pressure under typical atmospheric conditions,^4,5^ with a negative temperature dependence^6^ and reaction products dominated by formaldehyde (HCHO)^4,7^ and sulfur trioxide (SO_3_).^8,9^ Theory indicates that the reaction of an SCI with SO_2_ proceeds via the barrierless formation of a cyclic secondary ozonide (SOZ), followed by collisional stabilization of the SOZ or further rearrangement via one or more submerged barriers to form products including SO_3_.^10−15^ In the case of CH_2_OO + SO_2_, there is negligible stabilization of the SOZ under atmospheric conditions,^11,12^ and contributions from two stereochemical pathways lead to the production of HCHO + SO_3_ via submerged barriers.^12^ For reactions of other SCIs with SO_2_, there is greater potential for stabilization of the SOZ,^11^ leading to the potential for pressure-dependent kinetics and product yields.^11^

The atmospheric oxidation of SO_2_ by CH_2_OO is now expected to be limited, owing to competition with the rapid reaction of CH_2_OO with water dimers,^3^ but several field studies have demonstrated that significant discrepancies between measured and modeled concentrations of gas-phase sulfuric acid (H_2_SO_4_) in the atmosphere remain, which potentially result from incomplete consideration of the chemistry of SCIs other than CH_2_OO.^16−19^ Measurements in a boreal forest in Finland led to the suggestion that reactions of SCIs with SO_2_ may have been responsible for up to 50% of the H_2_SO_4_ observed in the gas phase,^16,17^ while observations in a rural location in Germany have indicated that SCI + SO_2_ reactions could be responsible for up to 80% of H_2_SO_4_ produced at night.^17^ Similarly, field experiments in Texas, United States, have suggested that nighttime production of H_2_SO_4_ is dominated by SCI + SO_2_ reactions, with potentially important contributions in the afternoon,^18^ and agreement between observations and model predictions for sulfate aerosol over the Southeast of the US has shown improvement when SCI + SO_2_ chemistry is included in the model.^20^ The role of SCI + SO_2_ chemistry in the atmosphere has also been investigated using observations made on Corsica, in the Mediterranean, where it was found that SCI + SO_2_ reactions could be responsible for 10% of the observed H_2_SO_4_ production during the day and 40% at night.^19^ Potential impacts of SCI reactions with SO_2_ have also been reported in vehicle exhausts^21^ and power plant plumes.^22^ However, there have been few measurements of the kinetics of SO_2_ oxidation by SCIs other than CH_2_OO, and uncertainties in rate coefficients for SCI + SO_2_ reactions have been highlighted as a constraint on our understanding of the atmospheric impacts of SCIs and the interpretation of field measurements of H_2_SO_4_.^16−20,23^

The SCI CH_3_CHOO exists in two conformers, *syn*-CH_3_CHOO and *anti*-CH_3_CHOO, which are separated by a significant barrier to interconversion (∼160 kJ mol^–1^)^24^ and thus behave as distinct species under ambient conditions.^25,26^ The first direct measurements of the reaction kinetics of CH_3_CHOO conformers, made using laser flash photolysis of CH_3_CHI_2_ in the presence of excess O_2_ coupled with tunable synchrotron photoionization mass spectrometry (PIMS), demonstrated rapid reactions with SO_2_ (R1 and R2).^25^
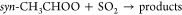
R1
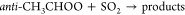
R2

The PIMS experiments were performed at 298 K and a total pressure of 4 Torr in He, giving *k*_1_ = (2.4 ± 0.3) × 10^–11^ cm^3^ s^–1^ and *k*_2_ = (6.7 ± 1.0) × 10^–11^ cm^3^ s^–1^. Formation of SO_3_ was observed, with a rate that suggested direct production from reactions of CH_3_CHOO conformers with SO_2_. Subsequent experiments using the PIMS technique at a fixed ionization energy gave a value for *k*_1_ of (1.7 ± 0.3) × 10^–11^ cm^3^ s^–1^ at 295 K and total pressures between 1 and 2.5 Torr in N_2_, with measurements indicating production of acetaldehyde (CH_3_CHO) from R1 at a yield of (0.86 ± 0.11) at 2 Torr.^5^ The reaction of *syn*-CH_3_CHOO with SO_2_ has also been investigated by monitoring the kinetics of OH radical production from the decomposition of *syn*-CH_3_CHOO occurring in competition with R1, giving *k*_1_ = (2.5 ± 0.2) × 10^–11^ cm^3^ s^–1^ at 298 K and 10 Torr in Ar.^27^

Experiments using laser flash photolysis of CH_3_CHI_2_/O_2_ mixtures with broadband UV absorption spectroscopy have also indicated that R1 and R2 are rapid.^26,28^ A rate coefficient of (2.0 ± 0.3) × 10^–11^ cm^3^ s^–1^ at 295 K and pressures between 7.5 and 500 Torr of N_2_ was reported from experiments in which the conformer-specific contributions to the total absorbance were not resolved.^28^ However, the result is expected to be dominated by *syn*-CH_3_CHOO on the basis of results from the earlier PIMS experiments,^25^ which indicated that *syn*-CH_3_CHOO represents 90% of the total CH_3_CHOO produced using the photolytic method. Conformer-specific measurements using broadband UV absorption spectroscopy have been achieved in experiments performed at 293 K and a total pressure of 10 Torr in He, giving *k*_1_ = (2.9 ± 0.3) × 10^–11^ cm^3^ s^–1^ and *k*_2_ = (2.2 ± 0.2) × 10^–10^ cm^3^ s^–1^.^26^ The conformer-specific UV experiments indicated that *syn*-CH_3_CHOO is the dominant conformer produced,^26^ in agreement with the earlier PIMS experiments,^25^ although a lower yield of 70% was reported, which may result from the different experimental conditions or uncertainties in the UV absorption cross-sections, particularly for *anti*-CH_3_CHOO.^26^

There are discrepancies in the literature for values of *k*_1_ and *k*_2_, but studies so far have all taken place at room temperature over a relatively narrow range of pressures (Table 1). Significant conformer dependence is shown for the reactivity of CH_3_CHOO with SO_2_,^25,26^ with studies also showing distinct conformer-dependent reactivity for reactions of asymmetric CIs with H_2_O^25,26,29^ and acids,^30^ as well as differences in their decomposition rates.^31^ CH_3_CHOO is the simplest Criegee intermediate that exists as two conformers and can therefore be used as a prototype to characterize the reactions of the larger CIs, which requires rate coefficients to be well established across a range of conditions. In this work, we report the kinetics of R1 and R2 at temperatures between 242 and 353 K and pressures between 10 and 600 Torr determined using time-resolved broadband UV absorption spectroscopy.

**Table 1:** Summary of Literature Results for *k*_1_ and *k*_2_^a^

reference	method	photolysis λ/nm	*T*/K	*p*/Torr	bath gas	[CH_3_CHI_2_]/10^13^ cm^–3^	[SO_2_]/10^13^ cm^–3^	*k*_1_/10^–11^ cm^3^ s^–1^	*k*_2_/10^–10^ cm^3^ s^–1^
Taatjes et al.^25^	LFP/PIMS	351	298	4	He		0.7–5	2.4 ± 0.3	0.67 ± 0.10
Smith et al.^28^	LFP/UV abs	248	295	7.5–500	N_2_	1300	155–600	2.0 ± 0.3	
Sheps et al.^26^	LFP/UV abs	266	293	10	He	1.5	0.8–4.8	2.9 ± 0.3	2.2 ± 0.2
Howes et al.^5^	LFP/PIMS	248	295	1–2.5	N_2_	1–10	2–9	1.7 ± 0.3	
Zhou et al.^27^	LFP/LIF	248	298	10	Ar	1.9–10.4	0.3–2.2	2.5 ± 0.2	

a LFP = laser flash photolysis, PIMS = photoionization mass spectrometry, UV abs = ultraviolet absorption.

## Experimental Section

The kinetics of R1 and R2 were studied as a function of temperature and pressure using laser flash photolysis of CH_3_CHI_2_/O_2_/N_2_/SO_2_ mixtures coupled with time-resolved broadband UV absorption spectroscopy. The experimental apparatus has been described in detail in previous work,^6,31^ and only a brief description is given here.

A dilute mixture of a known concentration of SO_2_ (Sigma-Aldrich, 99.9%) was prepared manometrically in N_2_ (BOC, 99.998%) and stored in a glass bulb before mixing in a gas manifold with N_2_ (BOC, 99.998%) and O_2_ (BOC, 99.5%) at known flow rates controlled by calibrated mass flow controllers (MKS Instruments). A known fraction of the total gas flow, controlled by a needle valve, was then passed through a bubbler containing liquid CH_3_CHI_2_ (SynHet, 90%) held at a constant temperature in a water bath before being recombined with the rest of the gas flow and passed into a jacketed Pyrex reaction cell. Experiments were performed under pseudo-first-order conditions, with the concentrations of SO_2_ in large excess over initial CH_3_CHOO concentrations. Concentrations were varied in the range [CH_3_CHI_2_] = (2.8–6.0) × 10^13^ cm^–3^, [O_2_] = (0.6–20) × 10^17^ cm^–3^, and [SO_2_] = (0.4–5.0) × 10^13^ cm^–3^, with typical initial [CH_3_CHOO] on the order of 10^12^ cm^–3^.

The reaction cell was 100 cm in length and 3 cm in diameter and sealed with fused silica windows at each end. The temperature of the cell was controlled by flowing liquid from a recirculating thermostatting unit (Huber Unistat 360) through the jacket surrounding the cell and calibrated by measuring the temperature of a flow of N_2_, under conditions identical to those used in kinetics experiments, at 5 cm increments along the length of the cell using a K-type thermocouple.^6,31^ Pressure in the cell was controlled by a rotary pump (EM2, Edwards) by throttling the exit to the reaction cell and measured by a capacitance manometer (MKS Instruments). The total flow rate through the cell was set to an equivalent of 1200 standard cm^3^ min^-1^ (sccm) at 50 Torr and adjusted with pressure to maintain a constant residence time in the cell of ∼2.6 s.

An excimer laser (KrF, Lambda-Physik CompEx 210) with output at λ = 248 nm and typical fluence of 30–40 mJ cm^–2^ was aligned along the length of the reaction cell using a dichroic turning mirror (Edmund Optics) and used to initiate production of *syn*- and *anti*-CH_3_CHOO in the cell via reactions R3 and R4a.
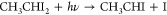
R3
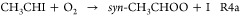
R4a
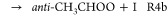
R4b

A delay generator (SRS DG535) was used to control the timing of the laser, which was operated with a pulse repetition frequency of 0.33 Hz to ensure that the gas mixture in the cell was replaced between each laser pulse.

Absorbing species in the cell were monitored by UV/visible radiation provided by a laser-driven light source (LDLS, Energetiq EQ-99X), which provided ∼10 mW cm^–2^ of light with a near constant radiance from 200 to 800 nm. The LDLS output was collimated by an off-axis parabolic mirror (ThorLabs) and aligned through the reaction cell in a multipass arrangement consisting of ten mirrors (Knight Optical), each of 12 mm diameter, resulting in an effective path length of (595 ± 53) cm for the experiments described in this work, which was determined using the method in our earlier work.^6^ Light exiting the cell was passed through a sharp cut-on filter (248 nm RazorEdge ultrasteep long-pass edge filter) to reduce the impact of scattered 248 nm light and focused into a fiber optic via a fiber launcher (Elliot Scientific). Light exiting the fiber optic was directed through a 25 μm slit onto a diffraction grating with 600 grooves/mm and imaged onto a thermoelectrically cooled charge-coupled device (CCD) detector (FER-SCI-1024BRX, Princeton Instruments). Photocharge generated on the CCD was shifted from an illuminated region to a storage region shielded from incoming radiation at set time intervals throughout the reaction, with the experimental setup used in this work giving a spectral resolution of ∼1 nm and a temporal resolution between 70 and 100 μs. Intensity data were typically recorded for 500 photolysis shots and transferred to a PC for analysis.

## Results

Absorbance spectra were determined from measured intensity data and related to the concentration of each species present using the Beer–Lambert law (eq 1)
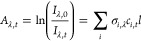
1where *A*_λ*,t*_ is the total absorbance at wavelength λ and time *t*, *I*_λ,0_ is the average pre-photolysis light intensity at wavelength λ, *I*_λ,*t*_ is the post-photolysis light intensity at wavelength λ and time *t*, σ_*i*,λ_ is absorption cross-section of species *i* at wavelength λ, *c*_*i,t*_ is the concentration of species *i* at time *t*, and *l* is the effective path length, which has a value of (595 ± 53) cm for experiments reported in this work.

**Figure 1:**
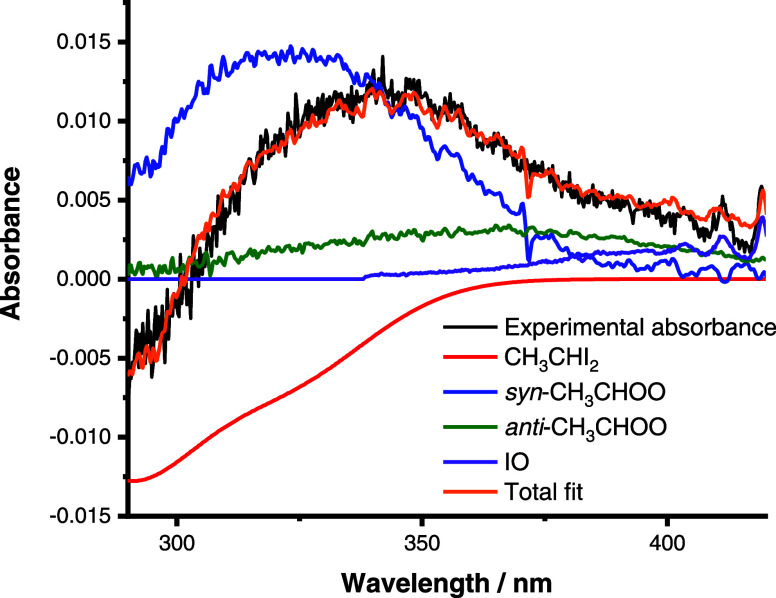
Observed absorbance (black), total fit (orange), and individual contributions from *syn*-CH_3_CHOO^26^ (blue), *anti*-CH_3_CHOO^26^ (green), CH_3_CHI_2_^32^ (red), and IO^33^ (purple) determined by fitting reference spectra to the observed absorbance using eq 1. For these data, *T* = 353 K, *p* = 10 Torr, *t* = 1 ms, [CH_3_CHI_2_] = 6.0 × 10^13^ cm^–3^. The fit to the observed absorbance for these data gave Δ[CH_3_CHI_2_] = (5.6 ± 0.07) × 10^12^ cm^–3^, [*syn*-CH_3_CHOO] = (2.3 ± 0.11) × 10^12^ cm^–3^, [*anti*-CH_3_CHOO] = (7.6 ± 0.27) × 10^11^ cm^–3^, and [IO] = (5.5 ± 0.14) × 10^11^ cm^–3^.

Figure 1 shows the typical absorbance measured following photolysis, which contains contributions from CH_3_CHI_2_, *syn*- and *anti*-CH_3_CHOO, and IO radicals produced by secondary chemistry within the system. Reference spectra for CH_3_CHI_2_,^32^*syn*- and *anti*-CH_3_CHOO,^26^ and IO^33^ were fit to the observed absorbance at each time point to determine the concentration of each species throughout the reaction. While absolute concentrations are reported here, it should be noted that uncertainties in the effective path length and absorption cross-sections do not contribute to uncertainties in measured kinetics for the pseudo-first-order conditions employed in this work.

**Figure 2:**
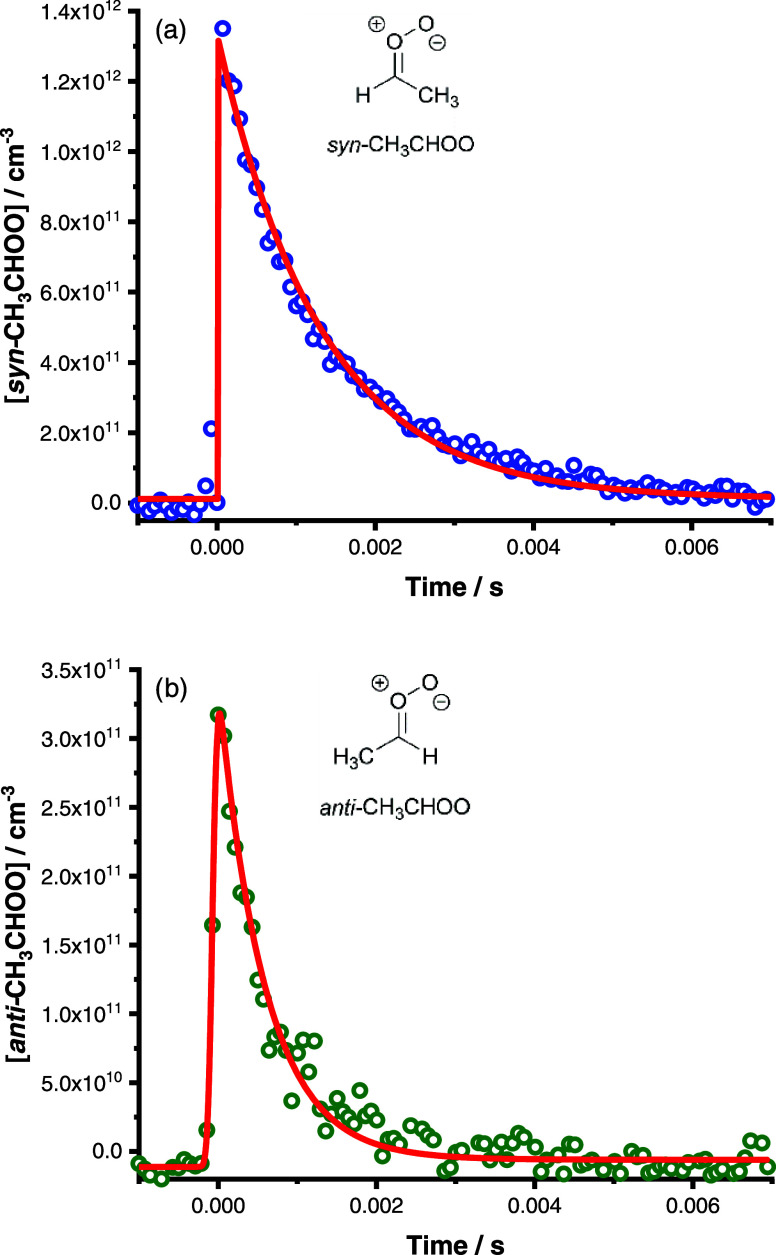
Observed concentration–time profiles for (a) *syn*-CH_3_CHOO and (b) *anti*-CH_3_CHOO. For these data, *T* = 298 K, *p* = 50 Torr, [SO_2_] = 1.1 × 10^13^ cm^–3^, and [CH_3_CHI_2_] = 2.8 × 10^13^ cm^–3^. The fits to eq 2 (coupled with the instrument response function as detailed in the Supporting Information) (solid lines) gave an initial concentration of (1.31 ± 0.03) × 10^12^ cm^–3^ and *k*′ = (765 ± 15) s^–1^ for *syn*-CH_3_CHOO and an initial concentration of (3.39 ± 0.02) × 10^11^ cm^–3^ and *k*′ = (2280 ± 218) s^–1^ for *anti*-CH_3_CHOO. Instrument response parameters were *w* = (2.99 ± 0.10) × 10^–5^ s and *t*_c_ = −(4.80 ± 0.09) × 10^–5^ s for both conformers. Uncertainties are 1σ.

Figure 2 shows the concentration–time profiles for *syn*- and *anti*-CH_3_CHOO in the presence of SO_2_, which were each fit according to a first-order kinetic loss (eq 2) convoluted with a Gaussian instrument response function (IRF) to describe the shifting of photocharge on the CCD detector (see the Supporting Information for further details).
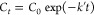
2where *C*_*t*_ is the concentration of *syn*- or *anti*-CH_3_CHOO at time *t*, *C*_0_ is the initial concentration of the Criegee intermediate conformer, and *k*′ is the rate coefficient describing the sum of first-order losses of the CH_3_CHOO conformer and is given by *k*′ = *k*_*x*_ + *k*_1_[SO_2_] for *syn*-CH_3_CHOO and *k*′ = *k*_*x*_ + *k*_2_[SO_2_] for *anti*-CH_3_CHOO, where *k*_*x*_ represents losses of *syn*- or *anti*-CH_3_CHOO via any reaction or process other than the reaction with SO_2_. Unimolecular decomposition and bimolecular reactions with the CH_3_CHI_2_ precursor contribute significantly to *k*_*x*_ for both *syn*- and *anti*-CH_3_CHOO,^31^ with potential additional contributions from reactions with iodine atoms, IO, or Criegee–Criegee chemistry as well as diffusion out of the probe region.

**Figure 3:**
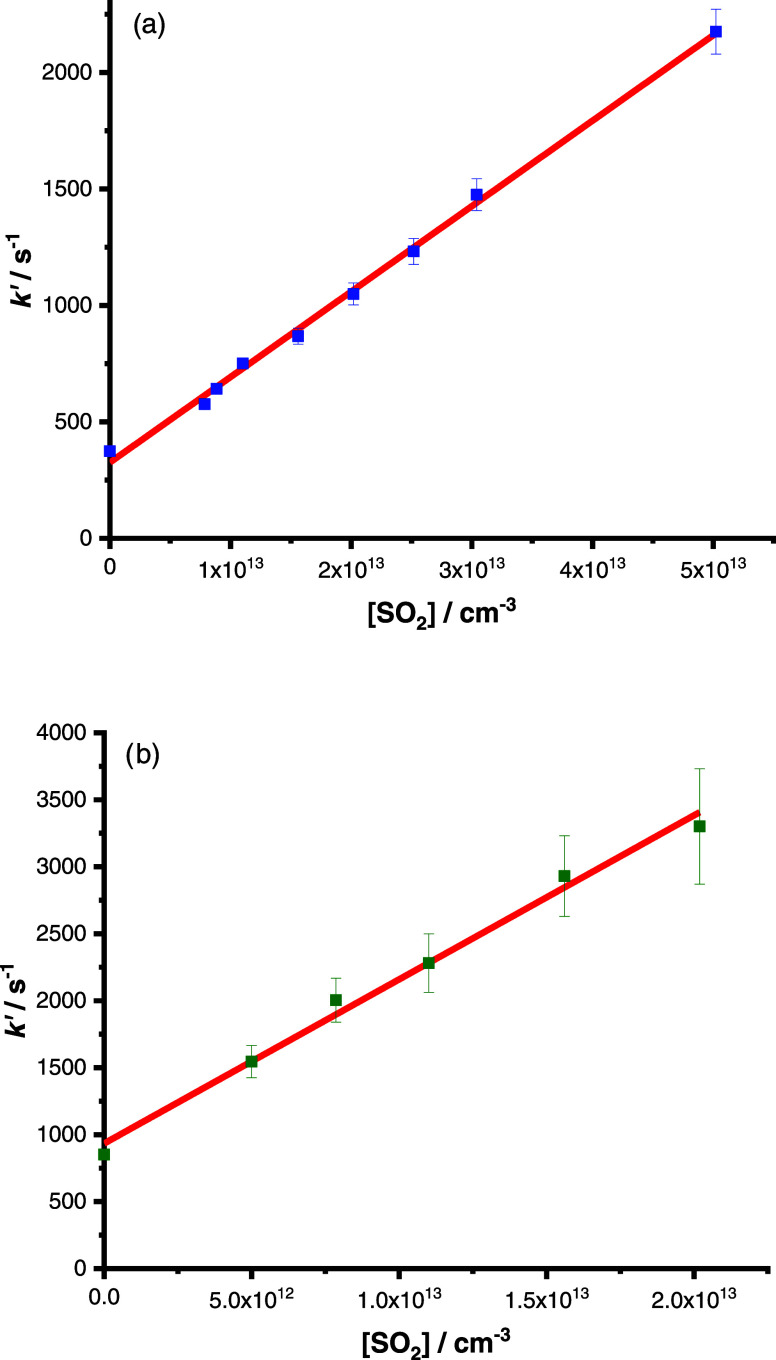
Dependence of *k*′ on [SO_2_] at *T* = 298 K and *p* = 50 Torr for (a) *syn*-CH_3_CHOO and (b) *anti*-CH_3_CHOO. Fits to the data (solid lines) gave *k*_1_ = (3.67 ± 0.07) × 10^–11^ cm^3^ s^–1^, with an intercept *k*_*x*_ of (326 ± 18) s^–1^, and *k*_2_ = (1.22 ± 0.10) × 10^–10^ cm^3^ s^–1^, with an intercept *k*_*x*_ of (934 ± 70) s^–1^. Uncertainties are 1σ.

Rate coefficients *k*_1_ and *k*_2_ were determined from the dependence of *k*_1_′ and *k*_2_′ on [SO_2_], respectively, with the typical results shown in Figure 3. Potential impacts of second-order losses for the CH_3_CHOO conformer through reactions such as CH_3_CHOO + CH_3_CHOO or CH_3_CHOO + I were also investigated by fitting concentration–time profiles to mixed first- and second-order kinetic losses convoluted with the IRF. Results for *k*_1_ and *k*_2_ obtained from the mixed-order fits were within 5% of those obtained from the first-order fits. Further details are given in the Supporting Information. All results reported here were obtained from first-order fits (eq 2).

**Figure 4:**
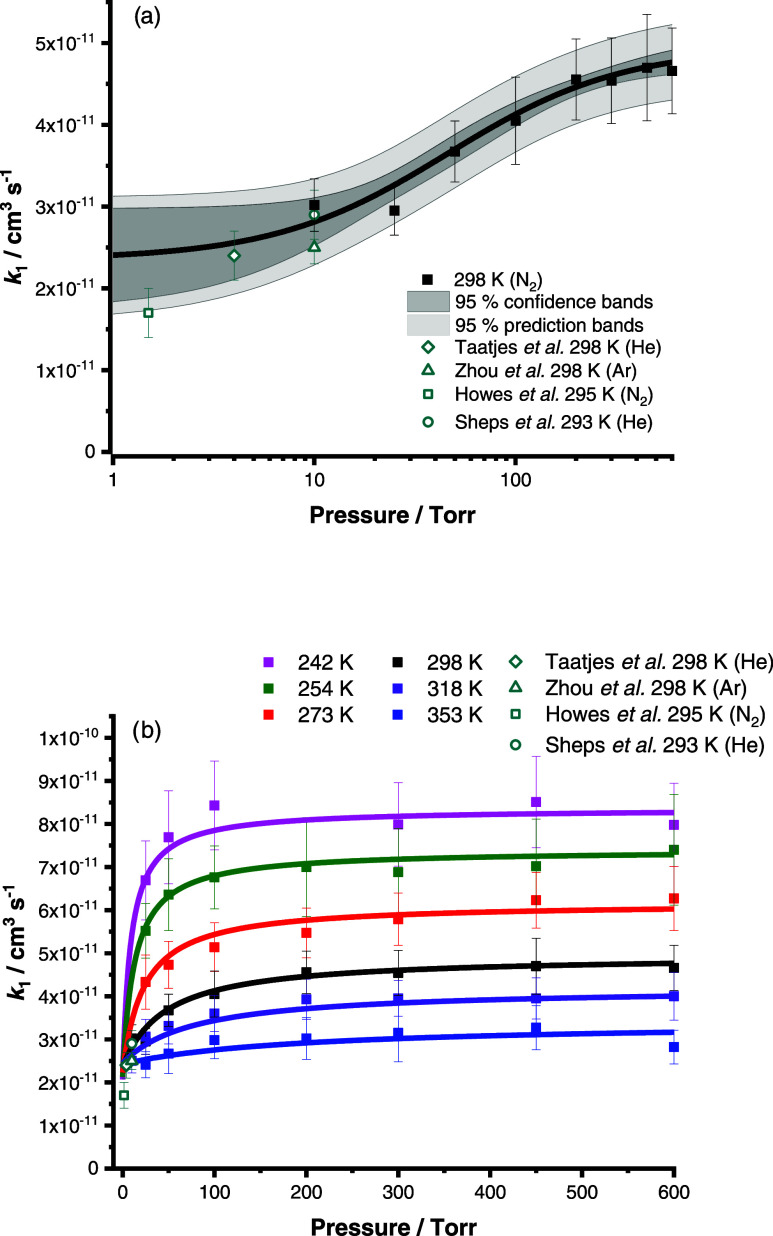
Effects of pressure on *k*_1_ at (a) 298 K and (b) all temperatures studied in this work. Solid lines show the fits to *k*_1_ using eqs 3–6 (which were performed globally using all data for *k*_1_ measured in this work). Previous results reported for *k*_1_ are also shown.^5,25−27^ Error bars represent a combination of the statistical error and the systematic errors resulting from uncertainties in gas flow rates and in the concentration of SO_2_.

Figure 4 shows the results for *k*_1_, which are summarized in Table 2. At 298 K, the results demonstrate an increase in *k*_1_ from (3.02 ± 0.32) × 10^–11^ cm^3^ s^–1^ at 10 Torr to (4.66 ± 0.52) × 10^–11^ cm^3^ s^–1^ at 600 Torr, where the uncertainties represent a combination of the statistical error and the systematic errors resulting from uncertainties in gas flow rates and in the concentration of SO_2_, with results at other temperatures also showing significant pressure dependence and overall negative temperature dependence. Equations 3–6, which describe a chemical activation mechanism with a non-zero rate coefficient at zero pressure,^34^ were fit globally to results obtained in this work for *k*_1_ over all temperatures and pressures to provide a parametrization for use in atmospheric models.
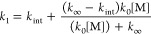
3where *k*_int_ represents the rate coefficient at zero pressure, *k*_0_ is the low-pressure limiting rate coefficient, and *k*_∞_ is the high-pressure limiting rate coefficient, and these are given by eqs 4–6
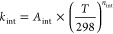
4
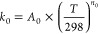
5
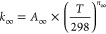
6Fit results gave *A*_int_ = (2.35 ± 0.39) × 10^–11^ cm^3^ s^–1^, *n*_int_ = (0.61 ± 0.79), *A*_0_ = (3.29 ± 1.30) × 10^–29^ cm^3^ s^–1^, *n*_0_ = −(9.52 ± 1.78), *A*_∞_ = (4.95 ± 0.51) × 10^–11^ cm^3^ s^–1^, and *n*_∞_ = −(2.52 ± 0.29).

**Table 2:** Summary of Results for *k*_1_ and *k*_2_^a^

*T*/K	*p*/Torr	[CH_3_CHI_2_]/10^13^ cm^–3^	[SO_2_]/10^13^ cm^–3^	*k*_1_/10^–11^ cm^3^ s^–1^	*k*_2_/10^–10^ cm^3^ s^–1^
**242**	25	3.0–4.1	0.6–3.1	6.69 ± 0.92	1.13 ± 0.13
	50			7.69 ± 1.08	1.17 ± 0.22
	100			8.43 ± 1.03	1.06 ± 0.17
	300			7.99 ± 0.97	1.06 ± 0.19
	450			8.51 ± 1.06	1.16 ± 0.25
	600			7.98 ± 0.97	
**254**	25	5.2–5.9	0.9–3.2	5.52 ± 0.63	0.90 ± 0.14
	50			6.36 ± 0.83	1.01 ± 0.20
	100			6.76 ± 0.73	1.00 ± 0.10
	200			7.00 ± 1.14	1.00 ± 0.18
	300			6.88 ± 1.02	
	450			7.02 ± 1.09	
	600			7.4 ± 1.28	
**273**	25	3.4–4.3	0.6–2.7	4.33 ± 0.63	1.29 ± 0.14
	50			4.73 ± 0.55	1.50 ± 0.28
	100			5.14 ± 0.57	1.54 ± 0.17
	200			5.47 ± 0.58	1.56 ± 0.18
	300			5.79 ± 0.61	
	450			6.23 ± 0.64	1.32 ± 0.21
	600			6.27 ± 0.74	
**298**	10	2.8–3.7	0.5–5.0	3.02 ± 0.32	1.04 ± 0.13
	25			3.00 ± 0.30	1.33 ± 0.17
	50			3.67 ± 0.37	1.22 ± 0.16
	100			4.05 ± 0.54	1.19 ± 0.17
	200			4.55 ± 0.49	1.08 ± 0.14
	300			4.54 ± 0.52	1.10 ± 0.20
	450			4.70 ± 0.65	1.26 ± 0.16
	600			4.66 ± 0.52	0.96 ± 0.16
**318**	10	4.1–5.3	0.7–4.1	2.50 ± 0.48	1.22 ± 0.22
	25			3.06 ± 0.41	1.42 ± 0.15
	50			3.31 ± 0.42	1.20 ± 0.21
	100			3.60 ± 0.42	1.05 ± 0.17
	200			3.93 ± 0.47	1.32 ± 0.22
	300			3.95 ± 0.42	1.41 ± 0.17
	450			3.95 ± 0.48	1.11 ± 0.20
	600			4.00 ± 0.56	1.08 ± 0.26
**353**	10	5.1–6.0	0.4–2.4		1.29 ± 0.20
	25			2.41 ± 0.30	1.09 ± 0.17
	50			2.67 ± 0.47	1.22 ± 0.25
	100			2.98 ± 0.43	1.10 ± 0.28
	200			3.02 ± 0.48	1.17 ± 0.19
	300			3.15 ± 0.67	1.13 ± 0.13
	450			3.27 ± 0.51	1.40 ± 0.25
	600			2.82 ± 0.39	0.88 ± 0.12

a Uncertainties represent a combination of the 1σ statistical error and the systematic errors resulting from uncertainties in gas flow rates and in the concentration of SO_2_.

The fits of eqs 3–6 indicate a value for *k*_1_ of (4.80 ± 0.46) × 10^–11^ cm^3^ s^–1^ at 298 K and 760 Torr. The pressure dependence observed in this work reconciles discrepancies between values for *k*_1_ reported at room temperature in previous work^5,25−27^ at pressures below 10 Torr, as shown in Figure 4. While kinetics reported by Smith et al.^28^ at 295 K over the pressure range of 7.5–500 Torr are in broad agreement with low pressure values for *k*_1_ reported in this work and in previous work, Smith et al. were unable to distinguish between the *syn*- and *anti*-conformers, so the rate coefficient reported will contain contributions from the reactivity of both *syn*-CH_3_CHOO and *anti*-CH_3_CHOO.

**Figure 5:**
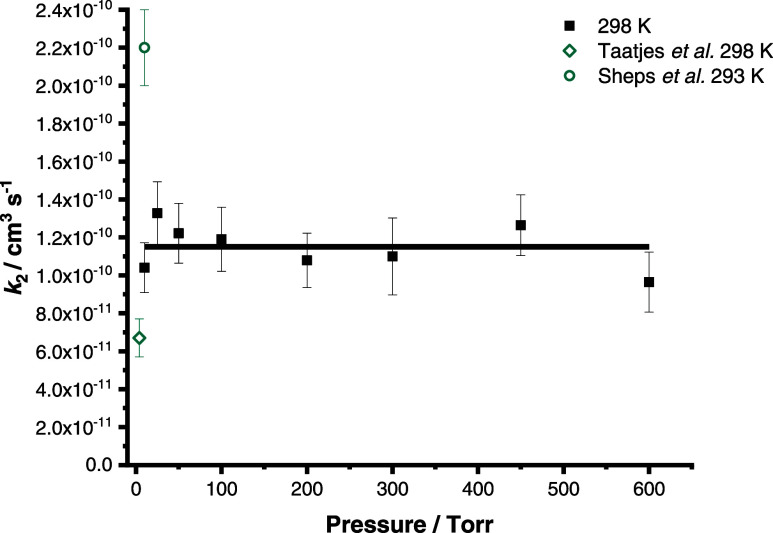
Effects of pressure on *k*_2_ at 298 K. The solid line shows the mean value for *k*_2_ at 298 K ((1.15 ± 0.16) × 10^–10^ cm^3^ s^–1^). Previous results reported for *k*_2_ are also shown. Error bars represent a combination of the statistical error and the systematic errors resulting from uncertainties in gas flow rates and in the concentration of SO_2_.

Figure 5 and Table 2 summarize the results obtained in this work for *k*_2_. In contrast to the results for *k*_1_, no significant dependence of *k*_2_ on the temperature or pressure was observed. At 298 K, results gave a mean value for *k*_2_ of (1.15 ± 0.16) × 10^–10^ cm^3^ s^–1^ between 10 and 600 Torr, with results over all temperatures and pressures giving a mean value of (1.18 ± 0.21) × 10^–10^ cm^3^ s^–1^. The effect of pressure on *k*_2_ at each temperature is shown in Figure S3 in the Supporting Information.

The kinetics of R2 have been reported in two previous studies^25,26^ at room temperature. Taatjes et al.^25^ performed experiments at 4 Torr using the PIMS technique and reported a value for *k*_2_ of (6.7 ± 1.0) × 10^–11^ cm^3^ s^–1^, while Sheps et al.^26^ performed experiments at 10 Torr using cavity-enhanced UV absorption spectroscopy and reported a value for *k*_2_ of (2.2 ± 0.2) × 10^–10^ cm^3^ s^–1^. Differences between the studies reflect the challenges associated with measuring such rapid kinetics, with the lack of dependence of *k*_2_ on temperature and pressure observed in this work potentially indicating that the kinetics for R2 are controlled by collision-limited or capture-limited kinetics. The difference in behavior between the *syn*- and *anti*-conformers is potentially influenced by lower steric hindrance for the *anti*-conformer, coupled with the higher ground state energy for *anti*-CH_3_CHOO by ∼15 kJ mol^–1^^24^ compared to *syn*-CH_3_CHOO and a higher dipole moment for *anti*-CH_3_CHOO than *syn*-CH_3_CHOO (5.53 D compared to 4.69 D, calculated at the B3LYP/AVTZ level of theory^35^).

**Figure 6:**
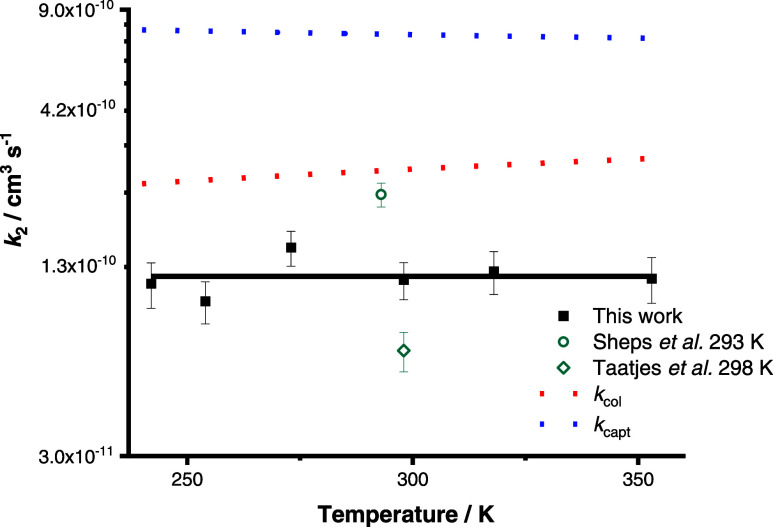
Mean values for *k*_2_ determined at each temperature. The solid line represents that the mean value for *k*_2_ over all conditions investigated in this work is (1.18 ± 0.21) × 10^–10^ cm^3^ s^–1^. Previous results reported for *k*_2_ and rate coefficients calculated using collision theory (*k*_col_, red dashed line) and capture theory (*k*_capt_, blue dashed line) are also shown. Error bars represent a combination of the statistical error and the systematic errors resulting from uncertainties in gas flow rates and in the concentration of SO_2_.

Figure 6 compares the experimental results for *k*_2_ with estimated values using a collision model (eq 7) and a capture model (eq 8).
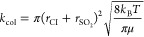
7where *r*_CI_ and *r*_SO_2__ are the effective radii of *anti*-CH_3_CHOO^36^ and SO_2,_^37^ respectively, *k*_B_ is the Boltzmann constant, *T* is the temperature, and μ is the reduced mass. The effective radius for *anti-*CH_3_CHOO was assumed to be the same as that reported in the literature for *syn*-CH_3_CHOO.^36^
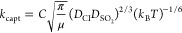
8where *C* is a constant (4.08 for the case of isotropic capture)^35,38^ and *D*_CI_ and *D*_SO_2__ are the dipole moments of *anti*-CH_3_CHOO^35^ and SO_2_,^39^ respectively.

The experimental results for *k*_2_ obtained in this work are lower than the estimated rate coefficients using either the collision model or the capture model, with experimental values a factor of ∼2 lower than those calculated from collision theory and a factor of ∼6 lower than those calculated from capture theory. However, the calculated values do offer some insight into the kinetics and suggest that R2 is close to the collision limit.

The reaction between *syn*-CH_3_CHOO and SO_2_ has been investigated using theoretical approaches, which indicate a barrierless reaction with a 98% yield of acetaldehyde (CH_3_CHO) + SO_3_ at 298 K and 200 Torr of He and a rate coefficient for CH_3_CHO + SO_3_ production of 4.49 × 10^–11^ cm^3^ s^–1^ at 298 K.^13^ However, the possible impacts of pressure were not fully discussed, and the reaction of *anti*-CH_3_CHOO + SO_2_ was not considered. The calculations^13^ predicted a positive temperature dependence for reactions of CH_2_OO, *syn*-CH_3_CHOO, and (CH_3_)_2_COO with SO_2_, despite the reactions being barrierless, and this is in contrast to the experimental results for *syn*-CH_3_CHOO + SO_2_ obtained in this work, our previous experiments for CH_2_OO + SO_2_,^6^ and experimental results for (CH_3_)_2_COO + SO_2_^40^ (see the Supporting Information for further details). Where potential impacts of pressure have been considered in detail in theoretical studies of SCI + SO_2_ reactions, there is an agreement with the lack of observed pressure dependence in the kinetics for CH_2_OO + SO_2_ under atmospheric conditions,^3,4,11,12^ but there are differences in the predicted pressure dependence of the reaction between (CH_3_)_2_COO and SO_2_.^11,12^ Vereecken et al. suggested that >80% of the SOZ formed by (CH_3_)_2_COO + SO_2_ undergoes prompt decomposition to acetone (CH_3_C(O)CH_3_) and SO_3_ at 298 K and a pressure of 4 Torr, while >97% of the SOZ collisionally stabilizes at 298 K and 760 Torr, with the difference compared to CH_2_OO + SO_2_ attributed to the greater number of degrees of freedom in the SOZ formed via (CH_3_)_2_COO + SO_2_, which would also be relevant to the comparison between the SOZ formed via CH_2_OO + SO_2_ and those from reactions of CH_3_CHOO conformers with SO_2_. However, Kuwata et al. calculated a different potential energy surface for the reaction between (CH_3_)_2_COO and SO_2_ compared to that reported by Vereecken et al., and thus a different mechanism for the reaction, with calculations predicting no significant collisional stabilization of the SOZ at 298 K and pressures below 10^4^ Torr and SO_3_ yields greater than 96% at 298 K and pressures from 1 to 760 Torr. Experimental measurements of the kinetics for (CH_3_)_2_COO + SO_2_ have indicated significant pressure dependence and negative temperature dependence under atmospheric conditions,^40−42^ similar to the observations in this work for the reaction between *syn*-CH_3_CHOO and SO_2_. Differences between theoretical approaches and between experiments and theory indicate that the application of theory to the prediction of SCI kinetics remains a challenge.

## Atmospheric Implications

**Figure 7:**
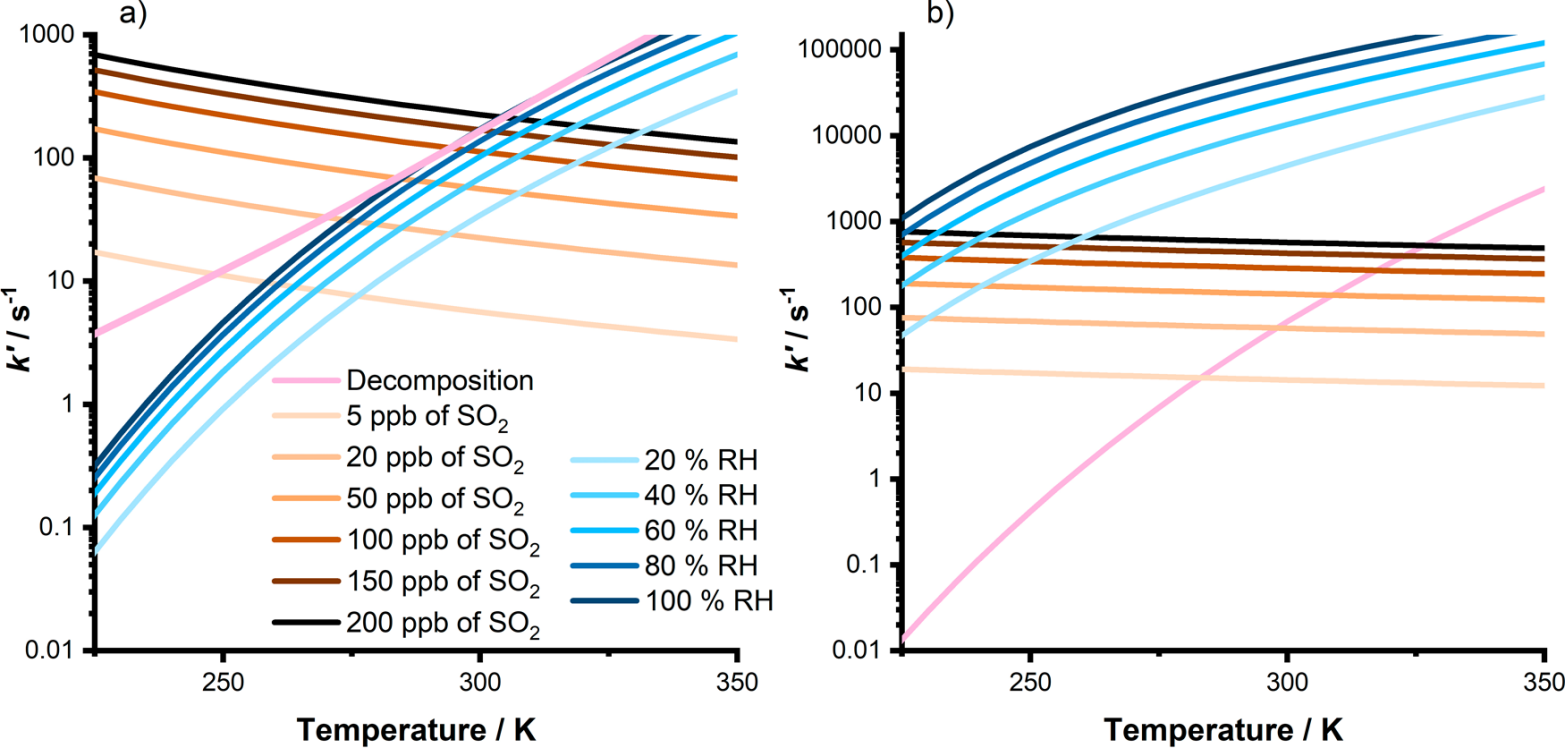
Pseudo-first-order losses of (a) *syn*-CH_3_CHOO and (b) *anti*-CH_3_CHOO through reaction with SO_2_ (orange), reaction with water vapor (blue), and unimolecular decomposition (pink) at a total pressure of 760 Torr. Pseudo-first-order losses were calculated as described in the main text.

The atmospheric impacts of CH_3_CHOO conformer reactions with SO_2_ depend on the competition with other CH_3_CHOO conformer reactions, which are expected to be dominated by unimolecular decomposition for *syn*-CH_3_CHOO and reaction with water vapor for *anti*-CH_3_CHOO.^31,43−45^Figure 7 compares the pseudo-first-order losses for CH_3_CHOO conformers through unimolecular decomposition and reactions with SO_2_ and water vapor for a range of SO_2_ and water vapor concentrations as a function of temperature at 760 Torr. Rate coefficients for unimolecular decomposition (*k*_dec_) were taken from our recent work,^31^ and those for reactions with SO_2_ (*k*_SO_2__) were taken from those determined in this work. Rate coefficients for reactions with water vapor (*k*_H2O_ and *k*_(H_2_O)_2__) were based on the upper limit for *syn*-CH_3_CHOO + H_2_O reported by Sheps et al. at 298 K,^26^ which forms the basis of the current IUPAC recommendation,^3^ and temperature-dependent measurements for *anti*-CH_3_CHOO + H_2_O and *anti*-CH_3_CHOO + (H_2_O)_2_ reported by Lin et al.^45^ Water dimer concentrations were calculated from the monomer concentration using equilibrium constants reported by Ruscic et al.^46^ There are no current reports of rate coefficients or upper limits for a possible reaction of *syn*-CH_3_CHOO with water dimers, and it should be noted that current IUPAC recommendations^3^ for *anti*-CH_3_CHOO reactions with water vapor do not extend beyond 298 K, owing to uncertainties in temperature-dependent measurements, which will impact the analysis shown in Figure 7. For *anti*-CH_3_CHOO, results show that the reaction with water vapor will dominate under all conditions relevant to the troposphere, but chamber studies employing high SO_2_ concentrations and low humidity will need to consider the impact of R2. For *syn*-CH_3_CHOO, the reaction with SO_2_ will be competitive with other losses in the atmosphere in areas with high SO_2_ concentrations and low humidity, particularly at low temperatures, contributing to the atmospheric oxidation of SO_2_. The pressure dependence of *k*_1_ indicates that there may be significant collisional stabilization of the SOZ produced in the reaction between *syn*-CH_3_CHOO and SO_2_, potentially limiting the production of SO_3_ and subsequently H_2_SO_4_. However, the fate of the SOZ is uncertain, and even if there is significant stabilization of the SOZ, it may still contribute to the atmospheric production of H_2_SO_4_ through subsequent chemistry. A more detailed assessment of the atmospheric impacts of CI reactions with SO_2_ would benefit from further experimental investigation of the nature and yields of the products, particularly as a function of pressure.

## Conclusions

The kinetics of *syn*- and *anti*-CH_3_CHOO reactions with SO_2_ have been investigated in the temperature range from 242 to 353 K at pressures between 10 and 600 Torr using laser flash photolysis of CH_3_CHI_2_/O_2_/N_2_/SO_2_ gas mixtures coupled with time-resolved broadband UV absorption spectroscopy.

Results for *syn*-CH_3_CHOO + SO_2_ show that the kinetics are pressure-dependent, with a negative dependence on temperature. The kinetics can be parametrized by a model that indicates a role for chemical activation, which gives a rate coefficient of *k*_1_ = (4.80 ± 0.46) × 10^–11^ cm^3^ s^–1^ at 298 K and 760 Torr. The observed pressure dependence reconciles apparent discrepancies in previous measurements of *syn*-CH_3_CHOO + SO_2_ kinetics performed at ∼298 K but at different pressures.

Kinetics of the reaction between *anti*-CH_3_CHOO and SO_2_ display no significant dependence on temperature or pressure over the ranges investigated. Results give a mean value for *k*_2_ of (1.15 ± 0.16) × 10^–10^ cm^3^ s^–1^ at 298 K and (1.18 ± 0.21) × 10^–10^ cm^3^ s^–1^ over all conditions studied in this work.

Comparisons with unimolecular decomposition kinetics of *syn*- and *anti*-CH_3_CHOO and reactions with water vapor under typical atmospheric conditions indicate that the reaction with SO_2_ will play an enhanced role in the removal of the *syn*-CH_3_CHOO in areas of low humidity and at low temperatures and the removal of *anti*-CH_3_CHOO is dominated by its reaction with water vapor under all conditions relevant to the troposphere.

## Supporting Information Available

Description of the instrument response function, comparison of first- and mixed-order analysis, effects of pressure on *k*_2_ across the studied temperature range, comparison to theoretical calculations, and summary of experimental data (PDF)
