# Supplementary material for: Kinetics of the Gas-Phase Reactions of syn- and anti-CH3CHOO Criegee Intermediate Conformers with SO2 as a Function of Temperature and Pressure
Source: J Phys Chem A. 2024 Mar 29;128(14):2815–24. doi: 10.1021/acs.jpca.4c00199 (PMC11017318; doi:10.1021/acs.jpca.4c00199)
Supplement: Supplementary file 1 — jp4c00199_si_001.pdf [file jp4c00199_si_001.pdf]

Kinetics of the Gas-Phase Reactions of *syn*- and *anti*-CH_3CHOO Criegee Intermediate Conformers with SO_2 as a Function of Temperature and Pressure

Rachel E. Lade,[1] Lavinia Onel,[1] Mark A. Blitz,[1,2] Paul W. Seakins,[1] and Daniel Stone[1*]

[1] School of Chemistry, University of Leeds, Woodhouse Lane, Leeds, LS2 9JT, UK

[2] National Centre for Atmospheric Science, University of Leeds, Woodhouse Lane, Leeds, LS2 9JT, UK

*Corresponding author: Daniel Stone d.stone@leeds.ac.uk

Supporting Information

Table of Contents

Instrument Response Function	Page S2
Comparison of First-Order and Mixed-Order Analyses	Page S3
Effects of Pressure on Kinetics of *anti*-CH_3CHOO + SO_2	Page S6
Comparison to Theoretical Calculations	Page S7
Summary of Experimental Conditions and Results	Page S9
References	Page S16

Instrument Response Function

Concentration-time profiles observed in this work are given by a convolution of the 'true' kinetic decay with an instrument response function (IRF) which results from the simultaneous illumination of multiple rows on the charge-coupled device (CCD) detector (see main text for details) and the row-by-row shifting of photocharge from the illuminated region of the CCD to a storage region. The IRF can be described by a Gaussian function with peak height *a* centred at t_c and with width *w* (Equation S1):

$$f(t) = a\ exp\left(-\frac{(t-t_c)^2}{2w^2}\right)$$

(Equation S1)

The 'true' kinetic decays for CH_3CHOO conformers under pseudo-first-order conditions are given by Equation S2:

$$C_t = C_0\ exp(-k't)$$

(Equation S2)

where C_t is the concentration of the CH_3CHOO conformer at time t, C_0 is the initial concentration of the CH_3CHOO conformer, and k' is the pseudo-first-order rate coefficient describing the loss of the Criegee intermediate.

Convolution of the IRF (Equation S1) with the first-order (or pseudo-first-order) kinetic decay (Equation S2) gives Equation S3:

$$C_t = \frac{C_0}{2}\ exp\left\{\frac{(k'w)^2}{2} - k'(t-t_c)\right\} \times \left\{1 + \mathrm{erf}\left(\frac{t-\ t_c - k'w^2}{\sqrt{2}w}\right)\right\}$$

(Equation S3)

where erf is the error function obtained in the integration of the normalised form of the Gaussian function.

Results given in the main text were obtained by fitting Equation S3 to the observed concentration-time profiles for *syn*- and *anti*-CH_3CHOO, with the IRF parameters t_c and *w* treated as global parameters.

The potential for mixedfirst- and second-order behaviour was also investigated (see below), in which case the 'true' kinetic decays are described by Equation S4:

$$C_t = \frac{C_0 k'}{k'\ exp(k't) - 2k''C_0 + 2k''C_0\ exp\ (k't)}$$

(Equation S4)

where C_t is the concentration of the CH_3CHOO conformer at time t, C_0 is the initial concentration of the CH_3CHOO conformer, k' represents the first-order (or pseudo-first-order) losses of the Criegee intermediate and k'' represents the second-order losses of the Criegee intermediate.

Convolution of the IRF (Equation S1) with the mixed first- and second-order kinetic decay (Equation S4) gives Equation S5:

$$C_t = \left\{\frac{1}{\left(\frac{1}{C_0} + \frac{2k''}{k'}\right)}\right\} \exp\left\{\frac{(k'w)^2}{2} - k'\ (t-\ t_c) + \frac{2k''}{k'}\right\} \times \frac{\left\{1 + \mathrm{erf}\ \left(\frac{t-t_c-k'w^2}{\sqrt{2}\ w}\right)\right\}}{2}$$

(Equation S5)

Investigation of potential mixed-order behaviour described below was performed by fitting Equation S5 to the observed concentration-time profiles for *syn*- and *anti*-CH_3CHOO, with the IRF parameters t_c and *w* treated as global parameters.

Comparison of First-Order and Mixed-Order Analyses

Concentration-time profiles for *syn*- and *anti*-CH_3CHOO were fit to both a first-order kinetic equation (Equation S2, coupled with the IRF to give Equation S3) and to a mixed first- and second-order kinetic equation (Equation S4, coupled with the IRF to give Equation S5) to investigate the potential impacts of any second-order reactions such as CH_3CHOO + CH_3CHOO or CH_3CHOO + I. For fits to the mixed-order equation, the second-order component (k'' in Equations S4 and S5) was treated as a global parameter for each conformer at each temperature and pressure.

Figure S1 shows a comparison between the first-order and mixed-order fits to typical concentration-time profiles for *syn*- and *anti*-CH_3CHOO at T = 298 K and p = 50 Torr, which indicates there were no significant differences between the first-order component obtained when describing the kinetics using a first-order model and when describing the kinetics using a mixed-order model.

Figure S2 compares rate coefficients for reactions of *syn*- and *anti*-CH_3CHOO with SO_2 obtained from the first-order fits to those obtained from the mixed-order fits. Results show less than a 5 % difference between the rate coefficients obtained when kinetics were described using the first-order and mixed-order models and values obtained for the intercept, k_x, between the two fits within their error limits. We therefore conclude that data are well described by pseudo-first-order kinetics.

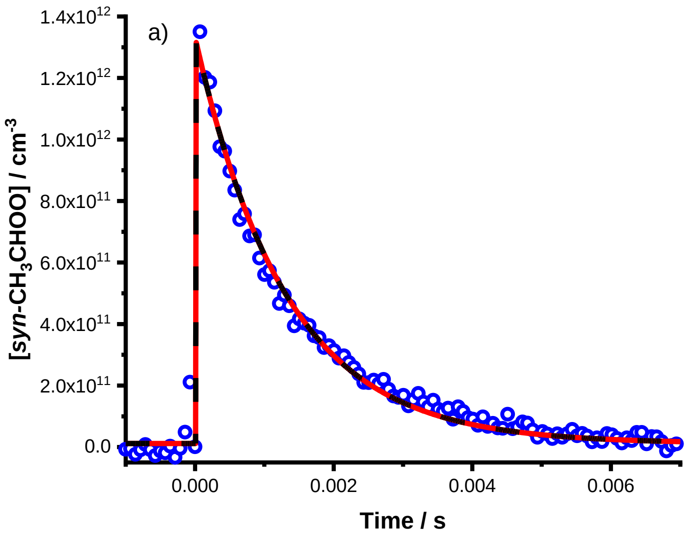

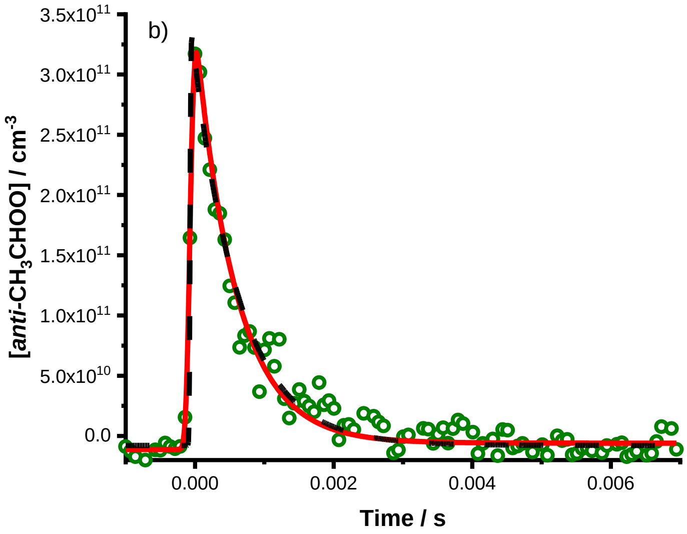

Figure S1. Comparison between first-order (solid red lines) and mixed-order (dashed black lines) for typical concentration-time profiles for a) *syn*-CH_3CHOO and b) *anti*-CH_3CHOO. The first-order fit to the *syn*-CH_3CHOO data gave k' = (765 ± 15) s^{-1} and $[CH_3CHOO]_0$ = (1.31 ± 0.03) × 10^{12} cm^{-3} and the mixed-order fit gave k' = (754 ± 15) s^{-1}, k'' = (7.66 ± 0.34) × 10^{-11} cm^3 s^{-1} and $[CH_3CHOO]_0$ = (1.35 ± 0.07) × 10^{12} cm^{-3}. The first order fit to the *anti*-CH_3CHOO gave k' = (2280 ± 220) s^{-1} and $[CH_3CHOO]_0$ = (3.39 ± 0.02) × 10^{11} cm^{-3} and the mixed-order fit gave k' = (2147 ± 93) s^{-1}, k'' = (2.55 ± 0.16) × 10^{-10} cm^3 s^{-1} and $[CH_3CHOO]_0$ = (3.47 ± 0.04) × 10^{11} cm^{-3}.

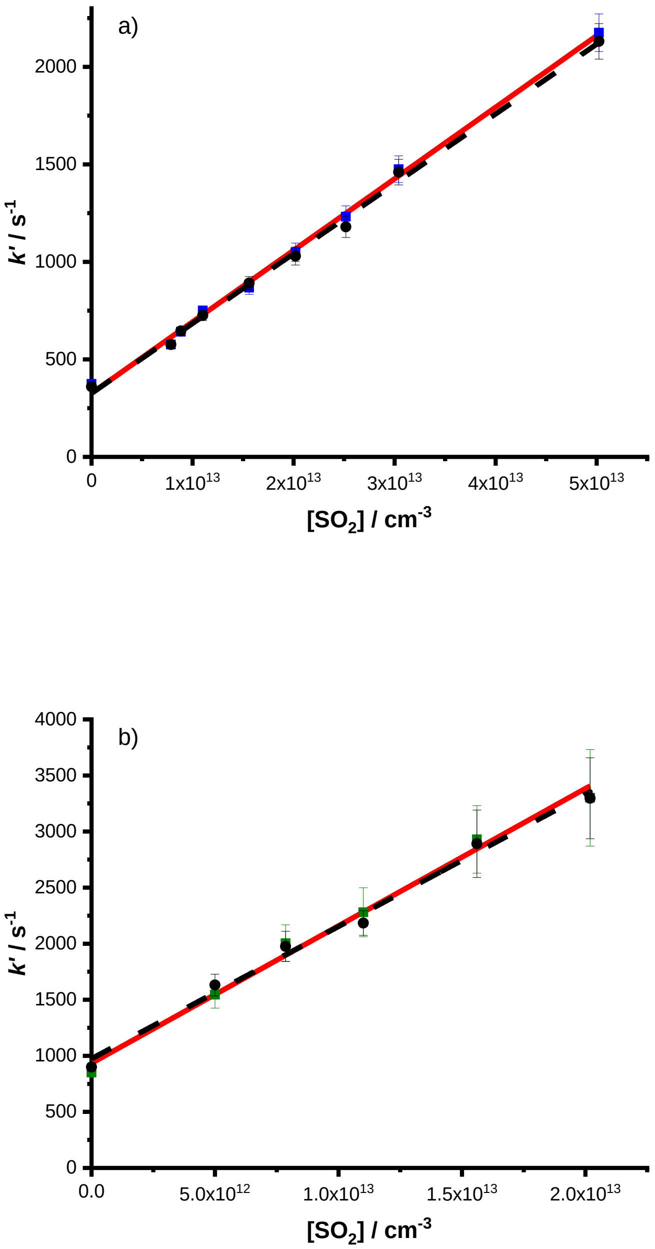

Figure S2. Dependence of *k′* on [SO_2] at T = 298 K and p = 50 Torr obtained for first-order fits (solid red lines) and mixed-order fits (dashed black lines) for a) *syn*-CH_3CHOO and b) *anti*-CH_3CHOO. Fits to the data for *syn*-CH_3CHOO gave k_1 (first-order, blue data) = (3.67 ± 0.07) × 10^{-11} cm^3 s^{-1}, with an intercept k_x of (326 ± 18) s^{-1} and k_1 (mixed-order, black data) = (3.58 ± 0.07) × 10^{-11} cm^3 s^{-1}, with an intercept k_x of (330 ± 9) s^{-1}. Fits to the data for *anti*-CH_3CHOO gave k_2 (first-order, green data) = (1.22 ± 0.10) × 10^{-10} cm^3 s^{-1}, with an intercept k_x of (934 ± 70) s^{-1} and k_2 (mixed-order, black data) = (1.17 ± 0.06) × 10^{-10} cm^3 s^{-1}, with an intercept k_x of (974 ± 67) s^{-1}. Uncertainties are 1σ.

Data reported in this work were obtained from the first-order fits.

Effects of Pressure on Kinetics of *anti*-$CH_3CHOO + SO_2$

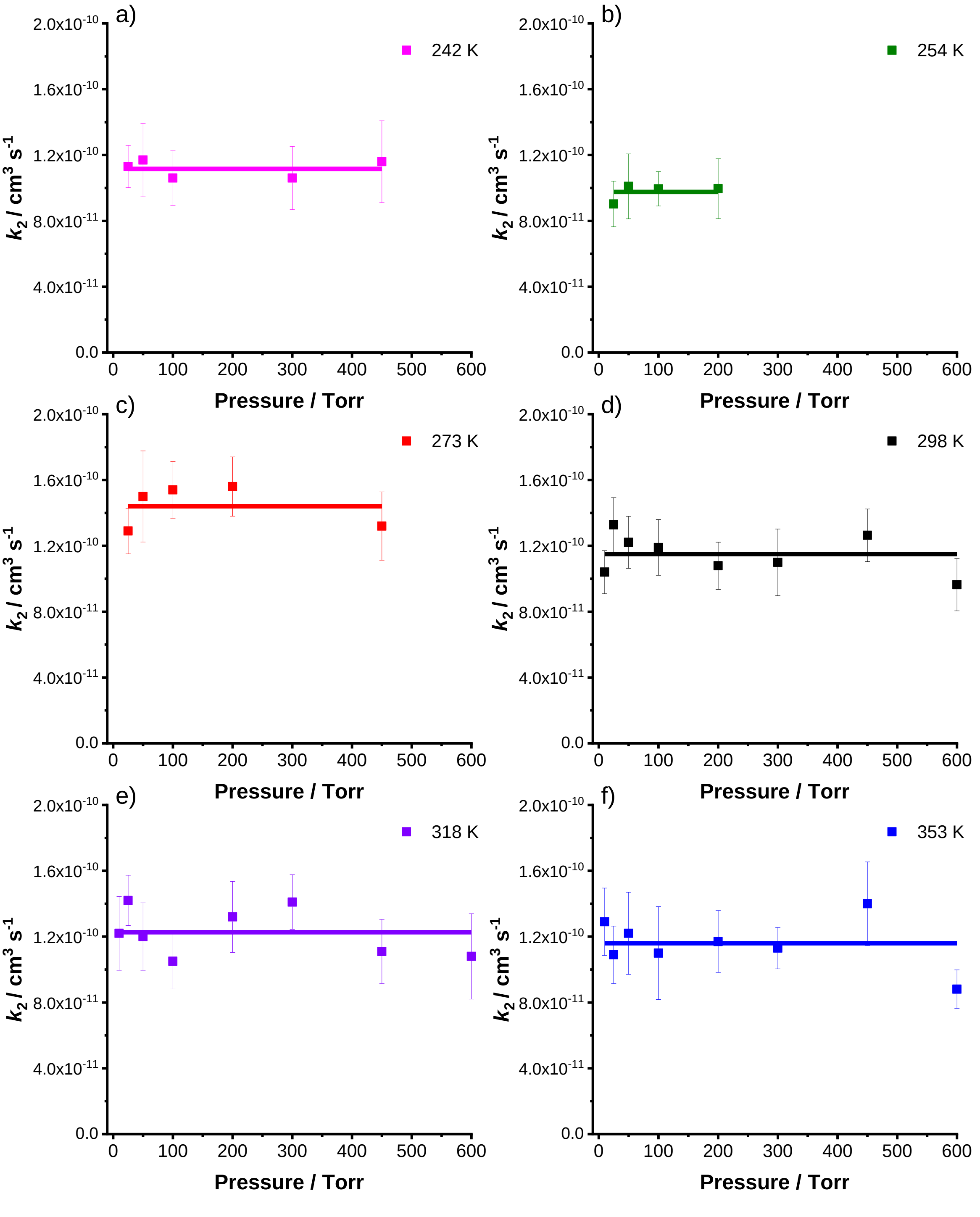

Figure S3. Effect of pressure on k_2 at each temperature, where solid lines represent the average value for the rate coefficient across each temperature. a) T = 242 K, k_2 = (1.12 ± 0.19) × 10^{-10} cm^3 s^{-1}; b) T = 254 K, k_2 = (0.98 ± 0.16) × 10^{-10} cm^3 s^{-1}; c) T = 273 K, k_2 = (1.47 ± 0.20) × 10^{-10} cm^3 s^{-1}; d) T = 298 K, k_2 = (1.15 ± 0.16) × 10^{-10} cm^3 s^{-1}; e) T = 318 K, k_2 = (1.23 ± 0.20) × 10^{-10} cm^3 s^{-1}; f) T = 353 K, k_2 = (1.16 ± 0.20) × 10^{-10} cm^3 s^{-1}.

Comparison to Theoretical Calculations

Theoretical calculations by Manonmani *et al.*[1] have predicted a positive temperature dependence for the reactions of CH_2OO , *syn*-CH_3CHOO and $(CH_3)_2CHOO$ with SO_2, in contrast to the negative temperature dependence shown in this work for *syn*-CH_3CHOO + SO_2, our previous work for CH_2OO + SO_2[2] and experimental results for $(CH_3)_2CHOO$ + SO_2.[3] Figure S4 shows a comparison between the temperature dependent results for CH_2OO, *syn*-CH_3CHOO and $(CH_3)_2CHOO$ with SO_2 reported by Manonmani *et al.* and the temperature dependent results of a) CH_2OO + SO_2 from our previous work,[2] b) *syn*-CH_3CHOO + SO_2 reported here and c) $(CH_3)_2CHOO$ + SO_2 using the IUPAC recommendation for the temperature dependence[4] (which is based on the work of Smith *et al.*[3]).

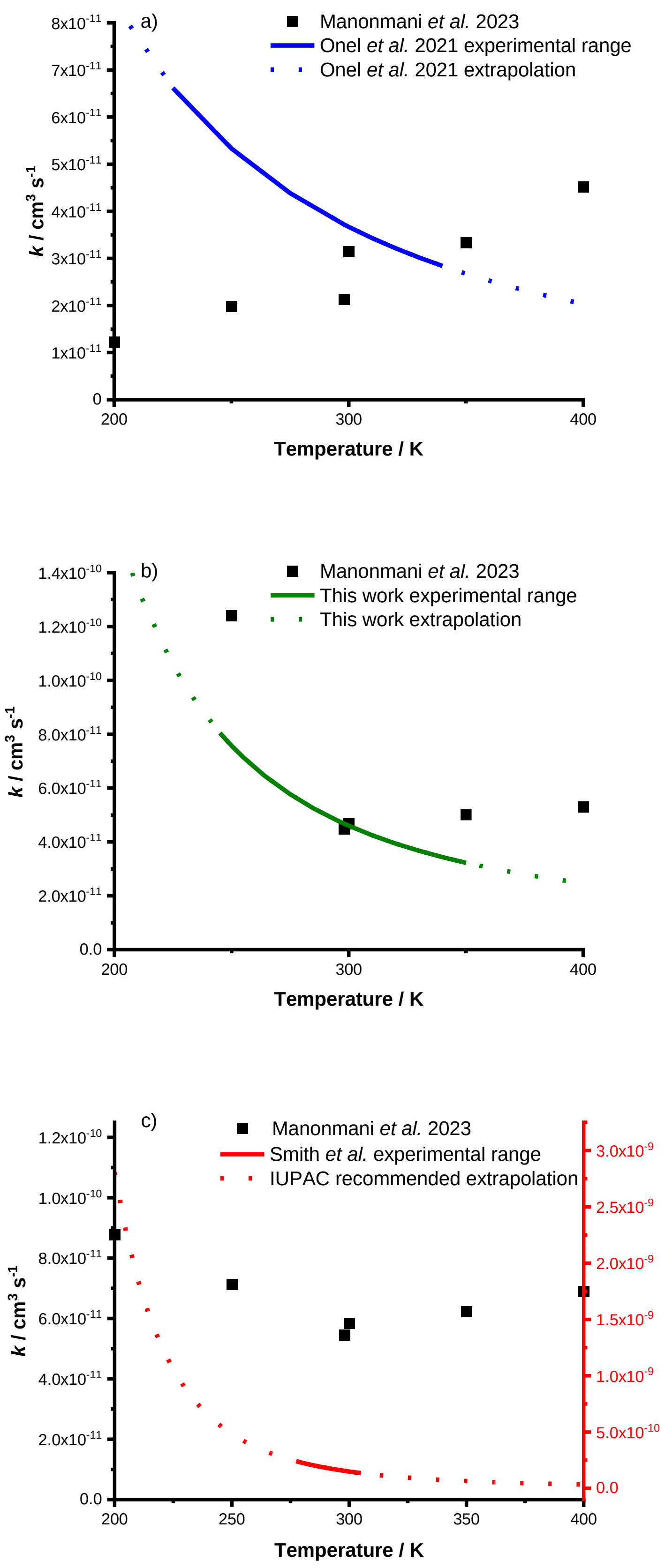

Figure S4. Comparison of rate coefficients as a function of temperature for a) $CH_2OO + SO_2$ reported by Manonmani *et al.*[1] and Onel *et al.*,[2] b) *syn*-$CH_3CHOO + SO_2$ reported by Manonmani *et al.* and the results of this work and c) $(CH_3)_2CHOO + SO_2$ reported by Manonmani *et al.* and the IUPAC recommended parameterisation.[4] The solid lines represent the experimental temperature range investigated in each study and the dotted lines are an extrapolation of the data to temperatures between 200 and 400 K.

Summary of Experimental Conditions and Results

Temperature / K	Pressure / Torr	$[SO_2]$ / 10^{12} cm^{-3}	k_1' / s^{-1}	k_2' / s^{-1}
**242**	25	0	790 ± 18	1454 ± 86
		5.8	1228 ± 24	2274 ± 130
		6.7	-	2165 ± 180
		7.5	1103 ± 31	2209 ± 178
		18	2048 ± 221	-
		22	2176 ± 126	3924 ± 575
	50	0	958 ± 13	2077 ± 89
		6.7	1234 ± 20	2910 ± 161
		7.5	-	2587 ± 385
		8.3	1393 ± 26	3225 ± 240
		11	1496 ± 47	3313 ± 413
		18	2282 ± 63	-
		22	2542 ± 66	-
	100	0	-	2031 ± 97
		6.7	1178 ± 23	2675 ± 172
		8.3	1198 ± 27	2758 ± 221
		13	-	3279 ± 583
		18	2037 ± 77	-
		22	2439 ± 23	-
	300	0	-	1062 ± 42
		5.9	957 ± 48	1651 ± 164
		6.7	1228 ± 30	1630 ± 82
		8.3	1322 ± 48	2296 ±210
		9.4	1258 ± 66	2414 ± 286
		11	1449 ± 69	-
		18	2159 ± 107	3566 ± 584
		26	2599 ± 97	3545 ± 465
	450	0	-	1419 ± 128
		7.1	1246 ± 29	2259 ± 196
		8.8	1587 ± 49	2427 ± 282
		9.9	1515 ± 46	2254 ± 282
		12	1638 ± 60	2981 ± 435
		19	2307 ± 94	-
		23	2697 ± 104	-
	600	0	970 ± 34	-
		7.1	1480 ± 50	-
		9	1475 ± 56	-
		14	1824 ± 54	-
		31	3425 ± 211	-
**254**	25	0	489 ± 18	886 ± 51
		8.5	808 ± 39	1895 ± 185
		11	1028 ± 52	2382 ± 278
		14	1225 ± 62	-
		31	2173 ± 127	3797 ± 822
	50	0	407 ± 18	343 ± 10

		8.5	-	585 ± 44
		12	848 ± 71	-
		14	1180 ± 79	1414 ± 159
		27	2108 ± 173	2954 ± 492
		31	2300 ± 165	-
	100	0	327 ± 15	1098 ± 62
		8.7	814 ± 29	1697 ± 141
		12	1004 ± 41	2211 ± 249
		14	1176 ± 48	2430 ± 266
		23	1962 ± 98	3331 ± 549
		28	2117 ± 106	3755 ± 618
		32	2430 ± 115	4240 ± 668
	200	0	593 ± 24	406 ± 31
		8.7	755 ± 41	1301 ± 159
		12	932 ± 83	-
		14	1090 ± 82	1246 ± 159
		23	2378 ± 208	-
		28	2470 ± 195	-
		32	2546 ± 152	3646 ± 727
	300	0	755 ± 28	-
		23	2187 ± 167	-
		27	2705 ± 160	-
	450	0	1162 ± 47	-
		9.6	1343 ± 59	-
		13	1410 ± 99	-
		16	1628 ± 103	-
		26	2624 ± 198	-
		31	3026 ± 210	-
		36	3415 ± 180	-
	600	0	641 ± 28	-
		10	918 ± 64	-
		17	1669 ± 141	-
		28	2250 ± 234	-
		33	3116 ± 272	-
**273**	25	0	653 ± 7	1691 ± 43
		6.6	893 ± 9	2488 ± 72
		7.3	834 ± 11	2627 ± 92
		8.6	861 ± 14	2813 ± 131
		17	1250 ± 31	-
		22	1616 ± 40	-
	50	0	417 ± 14	1536 ± 89
		6.1	557 ± 15	2206 ± 130
		6.7	636 ± 18	2421 ± 139
		7.6	640 ± 19	2884 ± 192
		7.9	647 ± 18	-
		10	1380 ± 49	-
		11	851 ± 24	3174 ± 201

		15	1047 ± 31	-
		24	1431 ± 47	-
	100	0	-	1208 ± 57
		6.6	537 ± 15	2169 ± 109
		7.3	641 ± 16	2382 ± 118
		8.3	567 ± 19	-
		8.6	654 ± 22	2528 ± 164
		12	866 ± 23	-
		17	1085 ± 41	-
		26	1549 ± 43	-
	200	0	-	1397 ± 66
		7.5	587 ± 24	2714 ± 330
		8.5	641 ± 26	2707 ± 330
		8.8	-	2742 ± 215
		13	906 ± 33	3392 ± 480
		17	1158 ± 40	-
		23	1423 ± 74	-
	300	0	453 ± 19	-
		6.7	795 ± 14	-
		7.4	793 ± 20	-
		13	1136 ± 38	-
		17	1345 ± 37	-
		23	1729 ± 62	-
		27	1992 ± 57	-
	450	0	-	1258 ± 70
		7.4	-	1849 ± 115
		8.3	811 ± 30	2206 ± 182
		9.7	870 ± 50	2529 ± 424
		14	1157 ± 60	-
		19	1444 ± 100	3715 ± 838
		25	1879 ± 119	-
	600	0	622 ± 27	-
		8.9	1048 ± 32	-
		15	1424 ± 82	-
		21	1736 ± 79	-
		32	2618 ± 228	-
**298**	10	0	384 ± 7	1071 ± 37
		8.9	570 ± 10	1477 ± 58
		10	625 ± 12	1532 ± 63
		15	794 ± 16	2267 ± 110
		20	946 ± 20	2851 ± 157
		25	1103 ± 24	3304 ± 202
		30	1249 ± 31	4042 ± 318
		8	532 ± 10	1465 ± 56
	25	8.9	665 ± 20	1086 ± 54
		10	723 ± 21	1358 ± 75
		15	856 ± 29	1729 ± 128

		20	1012 ± 32	2391 ± 178
		24	1166 ± 43	3261 ± 353
		30	1286 ± 44	-
		40	1581 ± 49	-
	50	0	374 ± 7	851 ± 35
		5	-	1546 ± 120
		7.9	576 ± 21	2004 ± 164
		8.8	642 ± 14	-
		11	765 ± 15	2280 ± 218
		16	868 ± 35	2930 ± 301
		20	1049 ± 47	3301 ± 431
		25	1231 ± 56	-
		30	1475 ± 68	-
		50	2175 ± 96	-
	100	0	470 ± 11	941 ± 47
		9	618 ± 17	-
		10	678 ± 19	1864 ± 124
		15	924 ± 27	-
		20	1139 ± 33	3306 ± 397
		25	1442 ± 46	1863 ± 121
		30	1568 ± 44	-
	200	8.2	725 ± 17	1353 ± 84
		8.9	786 ± 17	1314 ± 79
		10	847 ± 17	1550 ± 95
		15	1085 ± 24	2074 ± 158
		20	1336 ± 29	2329 ± 294
		25	1558 ± 38	3240 ± 345
		30	1692 ± 37	-
	300	0	601 ± 15	1363 ± 281
		9	876 ± 25	2138 ± 396
		10	928 ± 24	2329 ± 390
		12	1009 ± 29	2978 ± 565
		15	1159 ± 33	2812 ± 565
		20	1426 ± 33	4095 ± 712
		25	1734 ± 56	3745 ± 1013
		30	1855 ± 48	-
	450	0	652 ± 15	-
		8.1	-	1464 ± 163
		8.9	844 ± 24	1377 ± 158
		10	875 ± 24	1770 ± 210
		15	1112 ± 34	2209 ± 287
		20	1438 ± 41	2931 ± 442
		25	1806 ± 60	-
		30	1899 ± 52	-
	600	8.9	858 ± 39	1540 ± 395
		9.9	854 ± 34	1707 ± 372
		12	981 ± 44	2077 ± 419

		15	1181 ± 50	2531 ± 504
		20	1377 ± 55	2419 ± 471
		25	1658 ± 85	2956 ± 685
		30	1785 ± 73	3909 ± 832
**318**	10	0	710 ± 10	1121 ± 40
		9.5	898 ± 12	1641 ± 61
		11	991 ± 21	-
		13	1015 ± 20	2614 ± 152
		15	1030 ± 25	2209 ± 138
		17	1138 ± 24	-
		24	1299 ± 31	3276 ± 330
		33	1597 ± 37	5263 ± 439
		41	1665 ± 32	-
	25	0	647 ± 22	590 ± 31
		7	745 ± 26	1409 ± 74
		7.9	720 ± 22	-
		9.2	914 ± 26	1771 ± 90
		10	810 ± 26	1837 ± 101
		15	1047 ± 34	2362 ± 146
		21	1229 ± 34	3566 ± 236
		31	1510 ± 42	-
	50	0	-	322 ± 22
		7.1	668 ± 26	918 ± 55
		8	617 ± 23	990 ± 69
		9.3	808 ± 30	2111 ± 155
		10	849 ± 31	1941 ± 132
		15	960 ± 35	2429 ± 208
		21	1168 ± 43	-
		26	1224 ± 45	-
		31	1490 ± 54	3942 ± 347
	100	0	-	1497 ± 60
		6.8	1015 ± 34	1926 ± 126
		7.9	1048 ± 45	-
		9.2	1043 ± 50	-
		11	1127 ± 54	2059 ± 168
		15	1269 ± 57	2985 ± 261
		20	1430 ± 62	-
		25	1701 ± 82	4048 ± 465
	200	0	775 ± 15	669 ± 32
		6.8	-	1316 ± 50
		7.9	1043 ± 32	1608 ± 100
		8.6	1081 ± 39	-
		9.4	1156 ± 35	1648 ± 95
		14	-	2511 ± 146
		18	1420 ± 53	-
		23	1753 ± 69	-
		27	1794 ± 42	-

	300	0	692 ± 18	458 ± 29
		7.5	1065 ± 34	1304 ± 87
		9.2	1102 ± 35	1764 ± 139
		10	1118 ± 31	-
		15	1323 ± 40	2425 ± 184
		21	1521 ± 49	3409 ± 318
		25	1732 ± 74	-
	450	0	-	1635 ± 81
		7.4	1096 ± 19	-
		8.7	1289 ± 32	2268 ± 114
		9.4	1271 ± 38	2662 ± 166
		10	1354 ± 40	3097 ± 214
		15	1513 ± 39	2890 ± 165
		20	1667 ± 54	-
		25	1953 ± 71	4472 ± 370
		30	2042 ± 47	-
	600	0	913 ± 25	1286 ± 81
		6.7	-	1715 ± 146
		7.3	972 ± 26	-
		8.1	1064 ± 45	1691 ± 106
		9	1221 ± 68	1887 ± 154
		15	1500 ± 84	2972 ± 286
		20	1643 ± 77	-
		30	2006 ± 83	-
**353**	10	0	-	238 ± 26
		4	-	751 ± 39
		4.7	-	818 ± 42
		5.3	-	820 ± 47
		5.9	-	1079 ± 61
	25	0	1190 ± 43	0
		5.5	1263 ± 42	595 ± 27
		6.3	1368 ± 46	781 ± 35
		7	1305 ± 45	751 ± 35
		7.9	1377 ± 47	974 ± 46
		8.9	1335 ± 46	796 ± 37
		11	1445 ± 52	941 ± 46
		19	1618 ± 57	2099 ± 125
		22	1715 ± 59	-
	50	0	1545 ± 70	472 ± 26
		5.8	1522 ± 67	-
		6.6	1642 ± 78	922 ± 45
		7.4	1696 ± 81	-
		8.3	1627 ± 83	1418 ± 92
		9.4	1870 ± 95	1456 ± 102
		11	1803 ± 97	1910 ± 155
		20	2012 ± 100	-
		24	2115 ± 100	-

	100	0	1560 ± 52	641 ± 43
		6.1	1711 ± 74	1144 ± 73
		7.1	1840 ± 82	2001 ± 132
		8.2	1734 ± 77	1541 ± 96
		9	1738 ± 79	1804 ± 120
		10	1763 ± 81	2424 ± 165
		14	1804 ± 79	-
		19	2094 ± 98	2633 ± 173
		23	2375 ± 112	-
	200	0	1464 ± 43	889 ± 33
		6.4	1619 ± 83	1146 ± 80
		8.4	1763 ± 89	1377 ± 103
		9.1	-	1782 ± 142
		10	-	1723 ± 134
		14	1865 ± 103	2403 ± 190
		19	1898 ± 82	2965 ± 189
		27	2351 ± 155	-
	300	0	1854 ± 103	518 ± 42
		5.6	-	1047 ± 106
		7.1	1918 ± 114	-
		8	2022 ± 131	1303 ± 100
		9	-	1401 ± 86
		11	2072 ± 119	-
		19	2441 ± 122	2661 ± 197
	450	6.4	-	1695 ± 108
		7.2	-	1825 ± 100
		8.1	2101 ± 89	1955 ± 110
		9.1	2191 ± 99	-
		10	2237 ± 110	2027 ± 140
		14	2360 ± 108	2785 ± 206
		19	2486 ± 131	-
	600	6.7	2029 ± 149	1058 ± 106
		7.6	1957 ± 80	1312 ± 90
		8.5	-	1163 ± 52
		9.6	2032 ± 84	1393 ± 78
		11	2088 ± 86	-
		16	2261 ± 97	-
		23	2423 ± 119	2521 ± 211

References

1. Manonmani, G.; Sandhiya, L.; Senthilkumar, K., Reaction of Criegee Intermediates with SO_2–A Possible Route for Sulfurous Acid Formation in the Atmosphere. *ACS Earth and Space Chemistry* **2023,** *7* (10), 1890-1904.
2. Onel, L.; Lade, R.; Mortiboy, J.; Blitz, M. A.; Seakins, P. W.; Heard, D. E.; Stone, D., Kinetics of the gas phase reaction of the Criegee intermediate CH_2OO with SO_2 as a function of temperature. *Physical Chemistry Chemical Physics* **2021,** *23* (35), 19415-19423.
3. Smith, M. C.; Chao, W.; Takahashi, K.; Boering, K. A.; Lin, J. J.-M., Unimolecular Decomposition Rate of the Criegee Intermediate $(CH_3)_2COO$ Measured Directly with UV Absorption Spectroscopy. *The Journal of Physical Chemistry A* **2016,** *120* (27), 4789-4798.
4. Cox, R. A.; Ammann, M.; Crowley, J. N.; Herrmann, H.; Jenkin, M. E.; McNeill, V. F.; Mellouki, A.; Troe, J.; Wallington, T. J., Evaluated kinetic and photochemical data for atmospheric chemistry: Volume VII – Criegee intermediates. *Atmospheric Chemistry and Physics* **2020,** *20* (21), 13497-13519.
